# MambaPose: A Human Pose Estimation Based on Gated Feedforward Network and Mamba

**DOI:** 10.3390/s24248158

**Published:** 2024-12-20

**Authors:** Jianqiang Zhang, Jing Hou, Qiusheng He, Zhengwei Yuan, Hao Xue

**Affiliations:** 1 School of Electronic Information Engineering, Taiyuan University of Science and Technology, Taiyuan 030024, China; z2241007915@163.com (J.Z.); houjing101611@163.com (J.H.); xuehao202408@163.com (H.X.); 2 College of Modern Urban Construction Industry, Tianjin Chengjian University, Tianjin 300384, China; y19194042682@163.com

**Keywords:** pose estimation, Mamba, downsampling, feature fusion, loss function

## Abstract

Human pose estimation is an important research direction in the field of computer vision, which aims to accurately identify the position and posture of keypoints of the human body through images or videos. However, multi-person pose estimation yields false detection or missed detection in dense crowds, and it is still difficult to detect small targets. In this paper, we propose a Mamba-based human pose estimation. First, we design a GMamba structure to be used as a backbone network to extract human keypoints. A gating mechanism is introduced into the linear layer of Mamba, which allows the model to dynamically adjust the weights according to the different input images to locate the human keypoints more precisely. Secondly, GMamba as the backbone network can effectively solve the long-sequence problem. The direct use of convolutional downsampling reduces selectivity for different stages of information flow. We used slice downsampling (SD) to reduce the resolution of the feature map to half the original size, and then fused local features from four different locations. The fusion of multi-channel information helped the model obtain rich pose information. Finally, we introduced an adaptive threshold focus loss (ATFL) to dynamically adjust the weights of different keypoints. We assigned higher weights to error-prone keypoints to strengthen the model’s attention to these points. Thus, we effectively improved the accuracy of keypoint identification in cases of occlusion, complex background, etc., and significantly improved the overall performance of attitude estimation and anti-interference ability. Experimental results showed that the AP and AP50 of the proposed algorithm on the COCO 2017 validation set were 72.2 and 92.6. Compared with the typical algorithm, it was improved by 1.1% on AP50. The proposed method can effectively detect the keypoints of the human body, and provides stronger robustness and accuracy for the estimation of human posture in complex scenes.

## 1. Introduction

**Figure 1:**
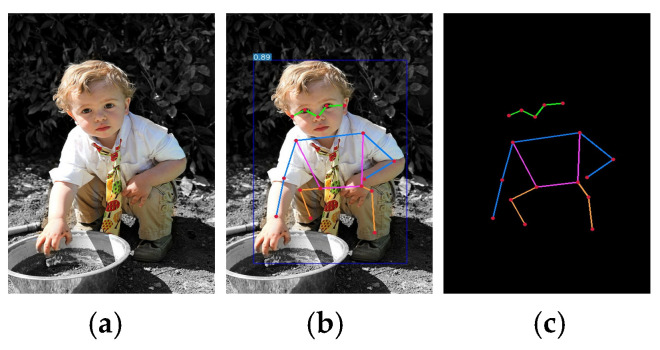
Detection results of MambaPose. (**a**) Input image; (**b**) test result graph; (**c**) posture image of the human body.

Human pose estimation is an important task in computer vision that aims to identify and locate keypoints in images or videos, such as joints and limbs. Pose estimation has a wide range of applications in security monitoring, motion analysis, human–computer interaction, virtual reality, and other fields. Accurate positioning of keypoints can help improve the understanding of intelligent systems and provide technical support for automatic analysis and decision-making for human activities. However, due to the diversity and complexity of human pose estimation, there are still problems of missed detection or false detection in dense scenes, and the accuracy of human detection of small targets is low. The results of our human pose estimation are shown in Figure 1.

Human pose estimation has undergone a multi-stage development from traditional methods to deep learning. It has gradually expanded from single-player to multiplayer scenarios, and has made significant improvements in real time and accuracy. Early human pose estimation methods mainly relied on hand-designed feature and probability models. These methods predict keypoint locations by extracting low-level visual features such as HOG [1] and SIFT [2] and combining them with statistical models such as the hidden Markov model (HMM) [3] or conditional random field (CRF) [4]. However, these methods are less adaptable to complex backgrounds or multi-person scenes and are not effective at occluding or overlapping human bodies. With the rise of deep learning technology, deep learning methods based on convolutional neural networks (CNNs) [5,6,7] have gradually become mainstream, which has brought revolutionary improvements to the accuracy and robustness of pose estimation. In 2014, DeepPose [8] was proposed to transform the pose estimation problem into a regression problem. The use of CNNs to directly predict the location of keypoints in the human body marks the first significant application of deep learning in this field.

Current deep learning methods are mainly divided into two strategies: top down and bottom up. The top-down approach first detects the human body region in the image through an object detection model [9], and then performs keypoint prediction for each human body. This method performs well in single-player scenarios, with high prediction accuracy. However, its computational complexity is high, especially in multi-person scenarios, where each detected human body needs to be processed individually, and its real-time performance needs to be further improved. In addition, if there is false detection or missed detection of human objects, it will directly affect the result of attitude estimation. A typical example is Mask R-CNN [10], which integrates human detection and pose estimation by adding keypoint branches. HRNet [11] further improves the prediction accuracy and maintains the delicacy of keypoint positioning through high-resolution features.

The bottom-up approach takes a global view and directly detects all keypoints and their connections in the entire image. These keypoints are then assigned to different human bodies by means of a mannequin or a human fitting algorithm. The advantage of this type of method is that it is more computationally efficient, especially in dense crowd scenarios, because it avoids the cropping and detection of human bodies one by one. However, the bottom-up approach is highly complex for post-processing, especially in crowded and heavily occluded scenes, where the assignment of keypoints can be incorrect. OpenPose [12] is an exemplar of the bottom-up approach, which for the first time realized the joint estimation of keypoints and connections in the human body by proposing the concept of part affinity fields (PAFs).

In recent years, with the growth in real-time application demand, the pose estimation algorithm based on the YOLO series has attracted extensive attention. Known for their efficient object detection performance, the YOLO family of models further improves the speed and usability of human pose estimation by integrating keypoint detection into their frameworks. YOLO-Pose [13] expands the keypoint prediction module on the basis of the YOLO architecture, integrates the attitude estimation task into the object detection process, and realizes end-to-end real-time prediction. This method not only retains the speed advantage of YOLO but also meets the accuracy needs of many real-world scenarios. In addition, Zhang et al. [14] introduced a lightweight ghost space pyramid pooling fast module to reduce the speed of model computation. A more efficient slim path aggregation network (Slim-PAN) module was proposed, which optimized the input relationship in the pyramid enhancement feature structure. Lu et al. [15] integrated the C2f feature extraction module with the KAN network on the basis of yolov8-pose, and introduced a nonlinear activation function to improve the feature extraction ability of the convolutional kernel. Although the YOLO-based method has significant advantages in real-time performance, it still faces challenges when dealing with small-target human bodies and dense multi-person scenes.

The motivation of our work is to solve the problems of missed detection and false detection in multi-person pose estimation in dense crowds, and at the same time to improve small-target detection accuracy for human beings. Recently, the state-space model (SSM)-based method has been successfully applied to image classification, medical segmentation, object detection, and other fields. In this paper, we explore the integration of the SSM algorithm and YOLO-Pose to adapt the application of the Mamba structure to human critical point detection tasks. Specifically, the main contributions of this paper are as follows.

Firstly, we combine the Mamba structure with YOLO-Pose and use local feature enhancement to make up for the problem of insufficient feature extraction of local information by the Mamba structure.

Secondly, we use slice downsampling instead of convolutional downsampling to enhance the selectivity for different information flow stages. Stitching four slice feature maps in the channel dimension can yield richer contextual information and spatial details.

Finally, we introduce adaptive threshold focus loss to improve the accuracy of keypoint recognition in cases of occlusion and complex backgrounds. A gated feedforward network is introduced to improve the detection ability of joint points in a small area.

## 2. Related Work

### 2.1. Top-Down Methods

The top-down method uses a human detector to select the position of each person in an input image, and after the box selects the human body, pose estimation is performed for each person. HRNet [11] proposed a deep high-resolution representation learning method to improve the accuracy of pose estimation by using high-resolution feature maps and deep networks to capture the details of human pose. Newell et al. [16] introduced the hourglass module and a multi-scale feature fusion mechanism to make full use of the multi-scale information in the image and improve the accuracy of human keypoint positioning. Chen et al. [17] proposed a method called cascade pyramid network (CPN), which aims to gradually refine the positioning of keypoints in the human body by constructing a multi-stage and multi-scale cascade architecture. Simple baselines [18], Mask-RCNN [10], and ViTPose [19] also adopt a top-down approach. The top-down approach typically relies on a human detector to generate bounding boxes, followed by keying within each bounding box. This method is highly dependent on the accuracy of the bounding box, and if the bounding box is not accurate, the key positioning will also be affected. In multiplayer scenarios, especially when multiple bodies occlude or overlap each other, a top-down approach is challenging. The process of locating keypoints within each bounding box is often independent and there is a lack of global contextual information, resulting in the model not being able to make full use of the information in the overall scene.

### 2.2. Bottom-Up Methods

The bottom-up approach predicts all body parts of each person in the input image, and then maps the human pose using mannequin fitting or other algorithms. The bottom-up approach does not rely on a separate human detector and directly predicts all the keypoints for everyone. Cao et al. [20] used convolutional neural networks (CNNs) to predict the body parts of all people in the image (e.g., head, shoulder, elbow, etc.), and then used the component affinity field to connect these parts into a complete human skeleton. Cheng et al. [21] proposed HigherHRNet, which significantly improved the accuracy and robustness of human pose estimation by introducing a multi-scale feature learning and global context information utilization mechanism. Geng et al. [22] introduced a decoupling keypoint regression mechanism, which divided keypoint detection and decoupling regression into two independent steps. Firstly, the keypoint detection is carried out on the feature map to generate the keypoint heat map. Then, the position of the keypoints is further optimized by the decoupling regression module. Luo et al. [23] redesigned the heatmap regression mechanism to make it more robust by introducing new loss functions and optimization strategies, especially when dealing with complex scenarios. The bottom-up approach requires the correct correlation of the detected keypoints to form a complete human skeleton. This process is especially difficult in multiplayer scenarios, where keypoints in different human bodies can be confused. Wrong associations can lead to incorrect pose estimation, especially in dense crowds or complex scenes.

### 2.3. State-Space Models

State-space models are an efficient sequence modeling technique for natural language processing (NLP) tasks. They solve the gradient-vanishing or explosion problem faced by traditional recurrent neural networks (RNNs) when dealing with long sequences by introducing structured state-space and linear complexity. Mamba [24] uses sparse convolution operations to simulate long-term dependencies in autoregressive models, thus avoiding the problem of gradient vanishing or exploding in traditional RNN. Mamba enables the calculation of linear time complexity by converting the input sequence into a specific representation so that the state of each position is only associated with a fixed number of pre-ordered states. Vision Mamba [25] is the first to apply the ideas of Mamba to the vision backbone network, achieving performance comparable to or even better than that of vision transformers (ViTs). This version of Mamba introduces the cross-scan module (CSM) to enhance the visual representation of the global acceptance domain by computational complexity in a linear fashion. Local Mamba [26] proposed a local scanning strategy to enhance feature dependence within the local window. VMamba [27] combines efficient selective scanning and backbone convolution to achieve a balance between accuracy and efficiency. VMamba further reduces the amount of computation by introducing a selective scanning mechanism that focuses only on the pre-order state, which has a significant impact on the current location. MSVMamba [28] is the latest version, and introduces a multiscale scanning mechanism that enhances the ability to learn dependencies between different resolutions. By applying the Mamba structure at different levels, MSVMamba enables the model to better capture the features of different scales, thereby improving the expressiveness of the model. Inspired by the remarkable effects of Mamba structures on other tasks, this paper proposes a MambaPose model to contribute to the field of human pose estimation for Mamba structures.

## 3. Methods

The purpose of human pose estimation is to locate and recognize the position of various keypoints of the human body in an image or video. In order to make up for the shortcomings of Mamba as the backbone network for local information modeling, we propose GMamba to extract keypoint information. Slice convolution is used for downsampling to avoid information loss caused by direct downsampling. We introduce a gated feedforward network to improve the detection of small targets. Adaptive threshold focus loss is introduced to dynamically adjust the weights of different keys.

### 3.1. Overview

**Figure 2:**
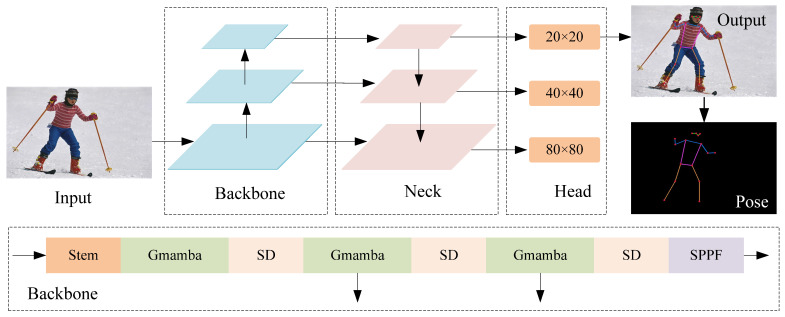
The overall structure of MambaPose. Our human pose estimation model consists of three parts: the backbone network, the neck, and the keypoint detection head. We can draw a posture map of the human body based on the keypoints of detection.

The architecture of MambaPose is shown in Figure 2. Our human keypoint detection model is divided into three parts: backbone, neck, and head. In the backbone network part, the input images are transformed for channel and dimension before the backbone extracts the features using Stem. Stem consists of a convolutional layer with a kernel of 3 × 3 and a step size of 2, a BN layer, and a SiLU activation function. Stacking the convolutional and batch normalization layers twice converts the dimensions of the input image h×w×3 to $\frac{h}{4}\times \frac{w}{4}\times c$. Then, the output feature map of Stem was entered into GMamba for feature extraction, and the output feature dimension was $\frac{h}{4}\times \frac{w}{4}\times c$. After slice downsampling, the image size is reduced by half, and the number of channels is doubled, and the dimension of the feature map becomes $\frac{h}{8}\times \frac{w}{8}\times 2c$. The downsampled feature map was entered into GMamba in the second stage to extract human features, and then slice downsampling was used for dimension transformation, and the size of the feature map was $\frac{h}{16}\times \frac{w}{16}\times 4c$. The steps of the second stage were repeated, and the dimension of the feature map obtained by downsampling in the third stage became $\frac{h}{32}\times \frac{w}{32}\times 8c$. Input into SPPF reduces the computational effort through the serial structure, while keeping the input and output feature map dimensions constant.

**Figure 3:**
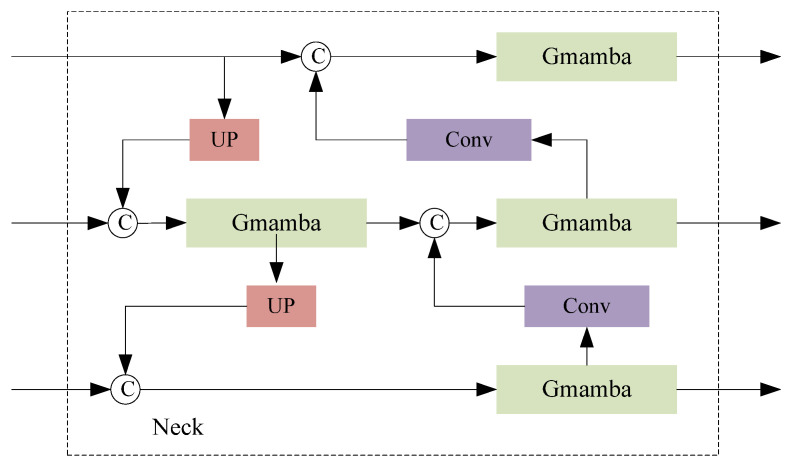
The overall structure of the neck. The neck uses the PA-FPN structure, and we use GMamba instead of C2F, which can effectively improve the information of keypoints in the human body during fusion. Top-down and bottom-up feature fusion methods are adopted, in which the convolutional layer is downsampled and the UP is upsampled.

In the neck, we use the design of PA-FPN to improve the network’s ability to capture contextual information and local information. The structure of the neck is shown in Figure 3, using the GMamba module instead of the traditional C2F module. GMamba can enhance the ability to express information in the process of feature fusion. By dynamically adjusting the spatial attention distribution, it can effectively highlight the important features related to the keypoints of the human body and suppress irrelevant or interfering information. We combine top-down and bottom-up strategies for feature fusion, which further enriches feature expression. In the top-down process, high-level semantic information is gradually fused through the layer-by-layer downsampling operation so as to retain the global context without losing key information. We add downsampling to the convolutional layer to reduce the feature map step by step by adjusting the size and stride of the convolutional kernel. In order to avoid excessive loss of feature details, we add the UP module to the upsampling operation to restore the resolution of the feature map by interpolation. The form of two pyramids improves the ability to detect keypoints in the human body.

### 3.2. GMamba

In the human pose estimation task, the selection of the backbone network is very important to the effect of feature extraction. Traditionally, ResNet [29,30,31] is often used as the backbone network for feature extraction, but its ability to model global information is weak and it is difficult to capture long-distance dependent features. This problem prompted the introduction of transformer [32,33,34,35], which leverages its advantages in modeling global context information to enable the network to effectively capture long-distance dependencies in the input feature map. However, the standard transformer has a high cost in terms of computational complexity, especially with high-resolution images, where the computational complexity is quadratic with the spatial dimension of the input feature map [36,37]. At the same time, the Mamba structure can be modeled over a long distance, which cannot effectively capture local information. To this end, we designed the GMamba structure to improve the ability to extract human features and reduce the computational complexity.

**Figure 4:**
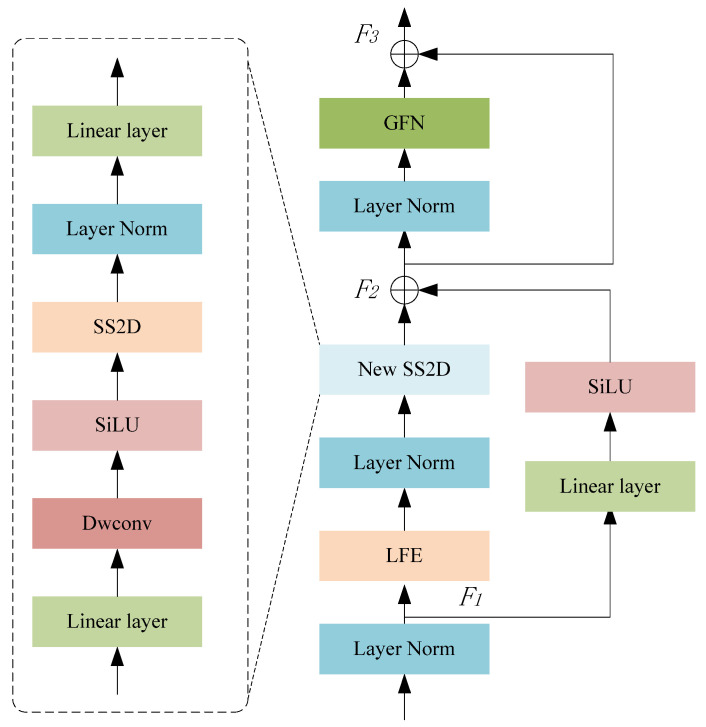
Overall structure of GMamba.

The overall structure of GMamba is shown in Figure 4. It includes gated feedforward networks (GFNs), local feature enhancement (LFE), and SS2D algorithms. In order to improve its detection ability for small targets, a gated feedforward network is added to the output of Mamba, and the gated feedforward mechanism controls the transmission of information by introducing gating signals so that the network can filter out interference information more flexibly. Since Mamba [24] can effectively carry out long-distance modeling, the ability to extract local information is insufficient, and local feature enhancement is used to strengthen the modeling effect of local information.

Mamba introduced the state-space model (SSM), which is a linear constant system function, to the field of image processing. Using a hidden state $h\left(t\right)\in R^{N}$ to map input $x\left(t\right)\in R$ to $y\left(t\right)\in R$, the mathematical expression of this system can be described as:

(1) $$h^{*}\left(t\right)=Ah\left(t\right)+Bx(t)$$

(2) $$y\left(t\right)=Ch\left(t\right)+Dx(t)$$

where x(t) represents the input sequence, $h\left(t\right)$ represents the hidden state, and $y\left(t\right)$ denotes the predicted output.

**Figure 5:**
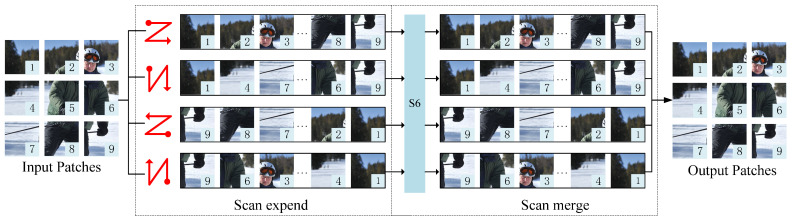
Schematic diagram of the workflow of SS2D. The scan expand operation expands the input image into a series of sub-images. Expand in four symmetrical directions: top-down, bottom-up, left, and right. Then, in the S6 block operation, the subgraph performs in-depth feature extraction. Finally, the extracted sub-images are merged into an output image of the same size as the input image by scanning merging. This process integrates information from all directions, and also ensures the spatial structure of the output feature map, which further enhances the network’s ability to understand and recognize human posture.

The SSM can efficiently handle one-dimensional sequential data, but the image information is non-sequential and contains spatial information. The SS2D algorithm [38] is introduced to solve this problem. SS2D employs three main steps: scan expansion, S6 block, and scan merging, and its overall flow is shown in Figure 5. For the input ${x\in R}^{h\times w\times c}$, the output obtained after GMamba can be described as:

(3) $$F_{1}=LN(x)$$

(4) $$F_{2}=\delta \left(LN(\psi (F_{1}))\right)+SiLU(LN(F_{1}))$$

(5) $$F_{3}=\beta \left(LN\left(F_{2}\right)\right)+F_{2}$$

where *LN* represents layer normalization, δ represents the SS2D operation, ψ represents the result after localized feature enhancement, and β represents the GFN operation.

#### 3.2.1. Gated Feedforward Network

**Figure 6:**
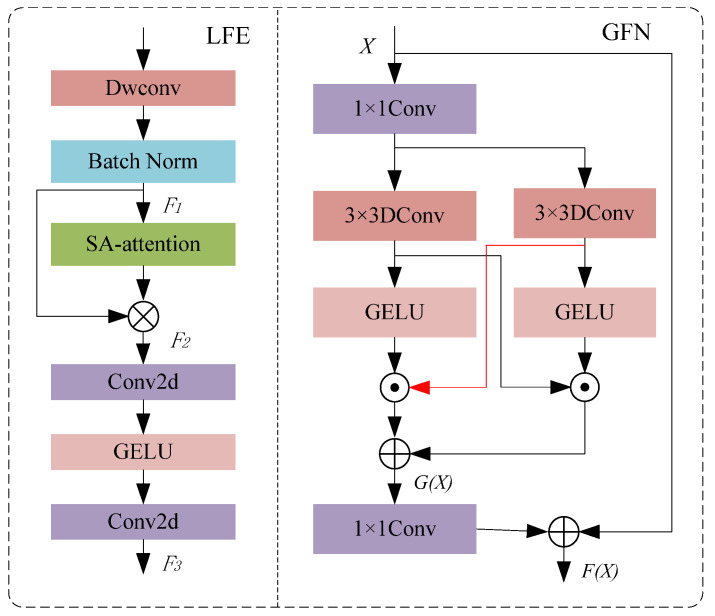
Overall structure of LFE and GFN.

In the human pose estimation task, a gated feedforward network (GFN) can be used to enhance the capture and representation ability of small target features. A diagram of GFN structure is given in Figure 6, which contains two parallel branches to effectively filter out features with less information and retain important feature details in the pose estimation process. GFN uses 1 × 1 convolution and 3 × 3 deep convolution operations to enrich local feature information: 1 × 1 convolution is used for information exchange and dimensionality reduction between channels, while 3 × 3 deep convolution is helpful for capturing finer spatial details. In the two branches, the dual GELU activation function is used to filter out the noise and irrelevant information by element-level products so as to retain only the features that are useful for pose estimation. This structure is particularly effective in detecting keypoints in small-target human bodies, as it enhances the feature expression of small target areas and prevents information loss. Useful information from the two paths is fused through an element-by-element additive operation to further improve the expressive ability of features. On each path, this structure enables GFN to better extract the local features of small targets in human pose estimation so as to improve the detection accuracy of small-scale joint points. The calculation process of GFN is as follows:

(6) $$G(X)=\omega (\alpha_{2}^{1}\left(\alpha_{1}^{1}\left(X\right)\right))⊙\alpha_{2}^{2}(\alpha_{1}^{2}(X))+\alpha_{2}^{1}\left(\alpha_{1}^{1}\left(X\right)\right)⊙{\omega (\alpha }_{2}^{2}(\alpha_{1}^{2}(X)))$$

(7) $$F\left(X\right)=\alpha_{1}G\left(X\right)+X$$

where $F\left(X\right)$ denotes the output of GFN, $\alpha_{1}$ and $\alpha_{2}$ represent 1 × 1 convolution and deep convolution, respectively, and ω denotes the GELU activation function.

#### 3.2.2. Local Feature Enhancement

The local feature enhancement module avoids the problem of loss of human body information. A diagram of LFE structure is shown in Figure 6. The input feature map is preliminarily processed using a 3 × 3 deep convolution and batch normalization operation. Deep convolution convolutes each input channel independently, effectively capturing the local information in the input feature map and reducing the amount of computation. Different from ordinary convolution, deep convolution can significantly extract spatial local information while maintaining computational efficiency. In addition, the batch normalization operation normalizes the convolution output, which stabilizes model training and accelerates convergence. The mathematical expression for the preliminary processing is as follows:

(8) $$F_{1}\left(X\right)=BN(\alpha_{2}\left(X\right))$$

where *BN* represents batch normalization and $\alpha_{2}$ represents deep convolution.

Then, the 1 × 1 convolution is used to calculate the attention weight map to generate a single-channel feature map, and each position represents the importance of the corresponding position in the input feature map. The attention weight map is normalized by the sigmoid function so that its value is between 0 and 1. The resulting attention map highlights the key information in the feature map while attenuating the interference of background noise, allowing the model to focus more on the target area. The attention weight map is multiplied by element by element and the convoluted feature map, and the weight of each pixel of the feature map is dynamically adjusted. In this way, the unimportant information can be effectively suppressed and the ability of the model to pay attention to key areas can be enhanced. The calculation process of $F_{2}$ can be expressed as:

(9) $$A=\sigma (\alpha_{2}(F_{1}(X)))$$

(10) $$F_{2}\left(X\right)=F_{1}\left(X\right)A$$

(11) $$F_{3}\left(X\right)=\alpha_{1}(\omega (\alpha_{1}(F_{2}\left(X\right))))$$

where *A* denotes the attention weight map, σ represents the sigmoid function, $F_{2}\left(X\right)$ represents the output of the attention mechanism, $\alpha_{1}$ represents the convolution operation, and ω represents the GELU activation function.

After the attention mechanism has been used to enhance the local features, the combination of point-by-point convolution and nonlinear activation function is used to further enrich feature expression. Point-by-point convolution is used to adjust the information interaction between channels and re-encode the feature map after the attention mechanism to make the channel features more expressive. The use of the GELU activation function further increases nonlinear expression ability to help capture complex feature patterns. Subsequently, the module restores the number of channels of the feature map to the number of input channels through a second 1 × 1 convolution operation so as to be consistent with the input feature dimension.

### 3.3. Slice Downsampling

An efficient downsampling strategy is introduced after the GMamba structure, which can avoid the loss of information caused by direct convolution downsampling. Slice downsampling (SD) uses a slice operation to slice the input feature map, selecting a sub-region of every other pixel and slicing it into four different sub-feature maps. The input image $x\in R^{h\times w\times c}$ is divided into sub-feature maps $x_{1}$, $x_{2}$, $x_{3}$ and $x_{4}$. Then, a 3 × 3 convolution operation is applied to each sub-feature map, with a convolution kernel size of 3 and a step length of 1, and the result of each slice after convolution is as follows:

(12) $$y_{i}=W\alpha (x_{i}),\;(i=1,\;2,\;3,\;4)$$

where $y_{i}$ denotes the convolution result of the *i*-th slice, W represents the convolution operator, and α represents the convolution operation.

The four feature maps of the convolution operation are stitched together in the channel dimension such that the feature maps contain richer context information and spatial details. The mathematical expression of the output of the spliced feature map can be described as:

(13) $$y=Contact(y_{1},y_{2},y_{3},y_{4})\in R^{\frac{h}{2}\times \frac{w}{2}\times 4c}$$

Subsequently, the spliced feature map is mixed by 1 × 1 convolution, and the batch normalization and SiLU activation function are used to further improve the expression and nonlinearity of the features. Slice downsampling can not only effectively compress the spatial dimension of the feature map but also ensure the computational efficiency and information integrity. The final output of the slice downsampling is mathematically expressed as:

(14) $$z=SiLU(BN(W_{PW}\alpha (y)))$$

where *SiLU* is the activation function, *BN* represents batch normalization, $W_{PW}$ represents the point-by-point convolution kernel, and the output $z\in R^{\frac{h}{2}\times \frac{w}{2}\times \mathrm{out}\_\dim}$ is the output feature map of downsampling.

### 3.4. Adaptive Threshold Focus Loss

Adaptive threshold focus loss is used in human pose estimation to solve the detection problem for small targets and joint overlap or occlusion. ATFL can adaptively adjust the loss weight such that the model pays more attention to difficult samples and small targets to effectively improve the accuracy of human pose estimation. In pose estimation, many joint points are small targets, and ATFL increases the model’s attention to small targets through the weighting of the focus mechanism to improve the detection effect of joint points. Adaptive threshold focus loss introduces a dynamic threshold τ, and the adaptive weight coefficient is calculated for each sample. When $p_{t}$ is closer to the threshold τ, the greater the weight of the loss. When $p_{t}$ moves away from the threshold, the weight decreases. The mathematical expression for ATEL is:

(15) $$L_{A}=-\alpha_{t}{\left(1-p_{t}\right)}^{\gamma }\left|p_{t}-\tau \right|\log\left(p_{t}\right)$$

where $p_{t}$ represents the predicted probability that the model belongs to the target category for the positive sample, $\alpha_{t}$ denotes the balance factor, and γ represents the focus factor.

## 4. Experiments

### 4.1. Settings

Implementation Details. Our model was deployed on an Ubuntu 22.04.4 LTS system using an Nvidia RTX 4090 graphics card. The environment of the model was based on Python 3.10 and PyTorch 2.1.0 for training and testing. We used random cropping, random rotation, random flipping, etc., for data augmentation. The SGD optimizer was used for optimization, the batch-size was 16, the input image size was 640 × 640, the learning rate was set to $1\times 10^{-2}$, the training image size was 640, and the model was trained in 500 epochs.

Datasets. We used the COCO 2017 dataset to train the human pose estimation model. The COCO 2017 dataset [39,40] is suitable for tasks such as object detection, semantic segmentation, human pose estimation, and image description generation. The image used for human pose estimation is marked with 17 keypoints to locate the position of each joint of the human body, and the posture features of the human body can be formed by connecting the relevant joints. Each key includes location coordinates and visibility flags, which are represented as (x, y, v), where x is the abscissa, y is the ordinate, and v is the visibility (v=0, 1, 2). We selected 56,599 images of human bodies in Train 2017 and 2364 images of pedestrians in Val 2017 as validation sets.

### 4.2. Evaluation Metrics

We used the common evaluation indicators of the COCO dataset and OKS-based measures for pose estimation. For quantitative evaluation, we used AP, AP50, AP75, APL, and AR to calculate the average accuracy and average recall for different target sizes. Higher values for AR indicate that the model is able to capture more human joints. We also added PCP, PDJ, and PCK to measure keypoint-detection accuracy and accurately and comprehensively evaluate the performance of the model.

### 4.3. Performance Evaluation

To verify the performance difference between the method in this paper and the current typical algorithms, we used both quantitative and qualitative methods to conduct comparison experiments. Typical algorithms for quantitative comparison include OpenPose, HRNet, Hourglass, YOLOv5s6-Pose, and YOLOv8x-Pose. The comparison experiments on the COCO2017 validation set are shown in Table 1. The MambaPose algorithm in this paper outperformed the current typical algorithms in all common performance metrics, and improved by 1.1% on AP50. Table 2 shows the comparative experiments of performance indicators such as model complexity. The PCK measurement in Table 2 was at the 0.5 threshold.

As can be seen in Table 2, the proposed algorithm performs better than other algorithms in terms of PCP, PDJ and PCK, and can effectively detect the keypoints of the human body. YOLOv8x-Pose is better than YOLOv8s-Pose on the PCP, PDJ, and PCK indicators, due to the large model and high computational complexity. The hourglass algorithm has the most network parameters, which is 277.8 M. The OpenPose algorithm has the most GFLOPs and the worst real-time performance. The algorithm in this paper has a large number of parameters and a long inference time, which can effectively detect small targets on the human body.

**Figure 7:**
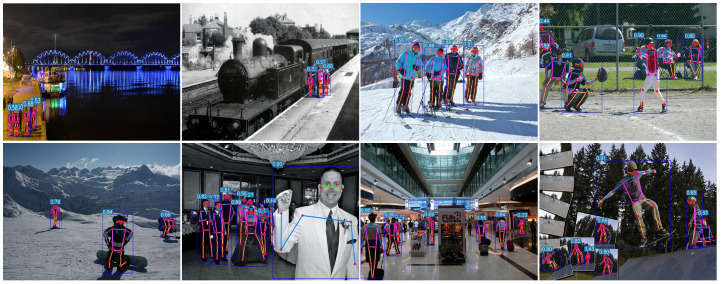
Visualization on the COCO 2017 validation dataset. MambaPose can effectively detect occluded human bodies and small human keypoints. The blue boxes indicate human bodies at different locations and the red dots indicate each joint point, using different-colored lines to connect adjacent joint points.

**Figure 8:**
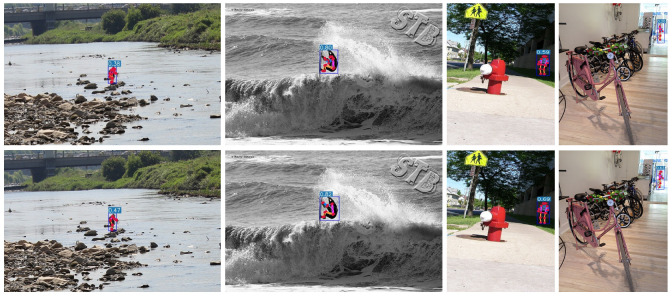
Comparison of the detection capabilities of small targets. Upper, results using YOLO-Pose; lower, results using the method in this paper.

**Figure 9:**
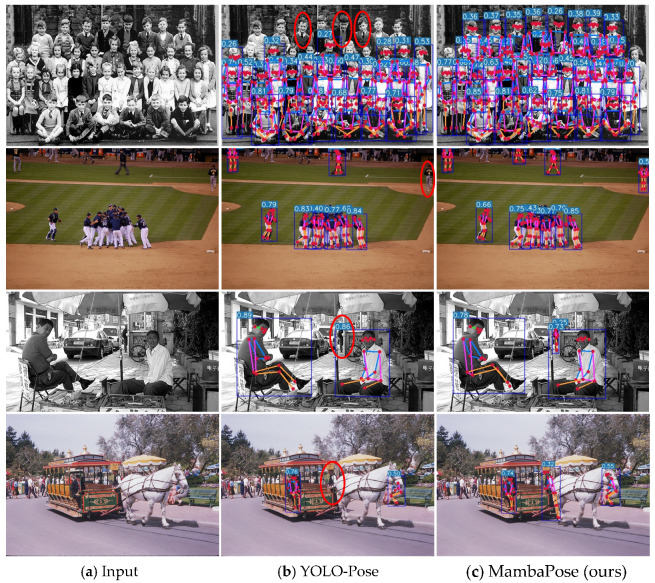
Visual rendering of missed detections in dense crowds. Red circles to mark the missing human bodies and keypoints.

**Figure 10:**
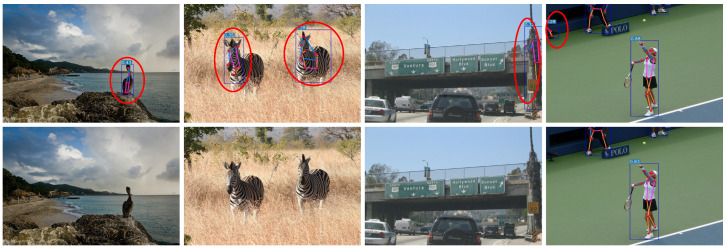
False detections in images. Upper, detection results using YOLO-Pose; lower, results using the method in this article. YOLO-Pose detects animals and other objects as pedestrians. Red circles indicate false detections.

Figure 7 shows the visualization of this paper’s algorithm on the COCO 2017 validation dataset. To verify the detection ability for small targets and dense crowds, we conducted a qualitative comparison with the current typical YOLO-Pose algorithm [13]. Figure 8 shows the comparative effects of small-target detection ability, and Figure 9 shows the comparative effects of detection ability for dense crowds. Figure 10 shows the results of false detection. In Figure 8, it can be seen that the algorithm of this paper improves the detection accuracy for small targets and can draw the human body posture more accurately. In Figure 9, it can be seen that YOLO-Pose has the problem of missed detection of human body parts, encapsulated in the red boxes. Our method can effectively improve the problem of missed detection in multi-person scenes.

As can be seen in Figure 10, the YOLO-Pose algorithm mistakenly detects other objects as human bodies. In the upper row, the first and second images misidentify animals as pedestrians and mark keypoints, while the third and fourth images falsely identify wires and a hand drill as human bodies. The algorithm in this paper reduced the false-detection rate and can effectively detect travelers and draw human postures.

### 4.4. Ablation Studies

#### 4.4.1. Ablation Experiments on MambaPose

To verify whether GMamba, slice downsampling, and gated feedforward in this paper can improve the detection accuracy of the keypoints of the human body, ablation experiments were conducted, and the results are shown in Table 3. Without GMamba indicates that the backbone uses Mamba for human feature extraction. Without slice downsampling means that downsampling uses a simple convolutional operation. Without GPN represents that GFN was directly removed in GMamba.

If we compare Model 5 and Model 6 in Table 3, it can be seen that GMamba improved 0.9 in AP compared to Mamba and that the GMamba proposed in this paper is feasible for human pose estimation. It effectively captured long-sequence information and localized information such that human joint information was better extracted. If we compare Model 4 and Model 6 in Table 3, it can be observed that the performance indexes of the slice downsampling strategy were improved compared with convolutional direct downsampling, and the spliced feature maps were enriched with contextual information and spatial details. Based on Model 3 and Model 6 in Table 3, the GFN extracted small targets better, and the overall performance metrics were improved.

#### 4.4.2. Ablation Experiments Using GFN in GMamba

We performed ablation experiments on the improved structure in order to test whether the introduced GFN could enhance the performance of the original Mamba structure inside GMamba. The GFN results using MLP, Conv MLP, Res-Conv MLP, and Gated MLP instead of GFN are shown in Table 4, in which it can be seen that the structure using GFN gave the best results for human pose estimation.

#### 4.4.3. Ablation Experiments on Adaptive Threshold Focus Loss

In ablation experiments with adaptive threshold focus loss, the effectiveness of each component was quantified by progressively removing or modifying the dynamic thresholds and focus factors of the ATFL and recording the model performance changes. The ablation experiments for the adaptive threshold focus loss function are shown in Table 5. If we compare Model 1 and Model 2 in Table 5, the introduction of adaptive thresholding made the model more flexible in focusing on difficult samples. Improving the recognition accuracy for complex backgrounds or diverse poses further enhanced the AP. In Model 3, the best ATFL performance was achieved with adaptive thresholding and focus factor. This indicates the weighted effectiveness of the focus factor on small targets or hard-to-detect regions, enabling the model to achieve more accurate recognition of critical regions in the pose estimation task.

## 5. Conclusions

In this paper, we propose a human pose estimation algorithm based on a gated feedforward network and Mamba, which mainly solves the problems of missed detection of keypoints and low accuracy of small-target human detection. We used the Mamba structure for a human pose estimation task, and made full use of it to effectively capture long-distance dependencies. However, Mamba is weak at modeling local information, so we designed a local feature enhancement module to strengthen the model’s ability to perceive local information of human features and locate keypoints more accurately. In addition, in order to further improve the small-target detection performance, we introduced a gated feedforward network optimized for the detection of small areas of human critical points. Experimental results showed that the proposed method performed well in multi-person scenarios and small-target human pose estimation tasks, not only improving the accuracy of keypoint detection but also outperforming the existing methods on both subjective and objective evaluation indexes. However, due to the large number of model parameters, it may face challenges in real time and in terms of limited computing resources in embedded device deployment. In the future, we will focus on further optimizing the network structure by reducing the number of model parameters and computational complexity. This will provide practical solutions for the deployment of embedded devices and promote the application and development of human pose estimation algorithms in practical scenarios.

## Figures and Tables

**Table 1:** Comparison experiments on the COCO2017 validation dataset.

Method	Backbone	Input Size	AP	AP50	AP75	APL	AR
OpenPose [12]	-	-	61.8	84.9	67.5	68.2	66.5
HRNet [11]	HRNet-W32	512	64.1	86.3	70.4	73.9	-
Hourglass [16]	Hourglass	512	56.6	81.8	61.8	67.0	-
YOLOv5s6-Pose [13]	Darknet_csp-d53	960	62.9	87.7	69.4	71.8	69.8
YOLOv8x-Pose [15]	-	640	71.7	91.5	78.4	78.3	77.9
MambaPose (ours)	GMamba	640	72.2	92.6	79.6	79.8	78.6

**Table 2:** Comparative experiments on performance indicators such as model complexity.

Method	PCP	PDJ	PCKh@0.5	#Params	GFLOPs	Inference Time
OpenPose [12]	0.52	0.59	81.6	-	451.1	36 ms
HRNet [11]	0.59	0.64	92.3	28.5 M	9.5	-
Hourglass [16]	0.43	0.51	79.2	277.8 M	-	-
YOLOv5s6-Pose [13]	0.67	0.67	93.5	15.1 M	20.5	9 ms
YOLOv8s-Pose [15]	0.69	0.71	94.8	11.6 M	30.2	5.1 ms
YOLOv8x-Pose [15]	0.78	0.74	95.1	69.4 M	263.2	9.3 ms
MambaPose (ours)	0.86	0.79	95.3	20.1 M	13.3	18 ms

**Table 3:** Ablation experiments of MambaPose structures.

Model	GMamba	Slice Downsampling	GFN	AP	AP50	AP75
1	x	√	x	68.3	89.4	70.5
2	x	x	√	69.7	89.8	71.3
3	√	√	x	70.5	90.7	72.1
4	√	x	√	71.6	90.9	74.4
5	x	√	√	71.3	90.8	73.5
6	√	√	√	72.2	92.6	79.6

**Table 4:** Results of ablation experiments using GFN in GMamba.

Model	AP	AP50	AP75
MLP	70.8	91.1	78.4
Conv MLP	71.1	91.6	78.5
Res-Conv MLP	71.5	91.5	78.3
Gated MLP	71.9	92.3	78.9
GFN (Ours)	72.2	92.6	79.6

**Table 5:** Ablation experiments with adaptive threshold focus loss function.

Model	Experimental Conditions	AP	AP50	AP75
1	Using a fixed threshold τ	71.6	89.9	78.3
2	Using adaptive thresholds τ (no focus factor γ)	71.9	90.7	78.8
3	Use ATEL (both adaptive thresholds τ and focus factors γ are used)	72.2	92.6	79.6

## Data Availability

The data generated during and/or analyzed during the current study are available from the corresponding author upon reasonable request.
